# Up-flow anaerobic sludge blanket bioreactor for the production of carboxylates: effect of inocula on process performance and microbial communities

**DOI:** 10.1186/s40643-025-00839-y

**Published:** 2025-01-24

**Authors:** Adrián Lago, Silvia Greses, Inés Moreno, Cristina González-Fernández

**Affiliations:** 1 Biotechnology Processes Unit, IMDEA Energy, Avda. Ramón de la Sagra 3, Móstoles, Madrid, 28935 Spain; 2 Thermochemical Processes Unit, IMDEA Energy, Avda. Ramón de la Sagra 3, Móstoles, Madrid, 28935 Spain; 3 Departament d’Enginyeria Química, CALAGUA-Unidad Mixta UV-UPV, Universitat de València, Avinguda de la Universitat s/n, Valencia, 46100 Spain; 4 Chemical and Environmental Engineering Group, ESCET, Rey Juan Carlos University, Móstoles, Madrid, 28933 Spain; 5 Department of Chemical Engineering and Environmental Technology, School of Industrial Engineering, University of Valladolid, Dr. Mergelina, s/n, Valladolid, 47011 Spain; 6 Institute of Sustainable Processes, Dr. Mergelina, s/n, Valladolid, 47011 Spain

**Keywords:** Anaerobic fermentation, Sugar cane molasses, Granular sludge, Carboxylates

## Abstract

This research investigated the acidogenic fermentation (AF) of sugar cane molasses in an up-flow anaerobic sludge blanket (UASB) reactor for the production of carboxylates. The first step was to assess the optimum process temperature (25, 35 or 55 ºC) using two different granular inocula, one from a brewery company (BGS) and other from a paper plant company (PGS). These experiments determined that the most suitable temperature for carboxylates production was 25 ºC, obtaining higher bioconversions (27.3 ± 0.3% using PGS and 39.2 ± 0.2% using BGS), despite the low pH value recorded (4.0-4.2). Then, both inocula were tested in UASB reactors. As a consequence of the operational conditions (25 ºC, pH = 5.5-6, organic loading rate (OLR) = 3 g_COD_·L^-1^·d^-1^ and hydraulic retention time (HRT) = 10 d), the microbial communities changed from those typical for biogas production to those specialised in the production of volatile fatty acids (VFAs). Indeed, the highest bioconversion efficiency (70.1%) was obtained with BGS, where uncultured *Eubacteriaceae* family microorganisms (56.0%) prevailed, enhancing the production of butyric acid (59.5 ± 2.4%w/w). Consequently, this inoculum was used to further identify the OLR threshold that should not be exceeded to attain optimal carboxylates production. OLR of 6 g_COD_·L^-1^·d^-1^ resulted in a decrease in bioconversion efficiency (59.5%). The VFAs pool was dominated by butyric acid (63.0 ± 1.4%w/w at an OLR of 4.5 g_COD_·L^-1^·d^-1^ and 52.8 ± 2.2%w/w at 6 g_COD_·L^-1^·d^-1^). The microbial community became even more specialised, increasing the presence of Firmicutes and Actinobacteriota phyla, proving that the imposed conditions favoured the production of VFAs when operating semicontinuously fed UASB reactors.

**Supplementary Information:**

The online version contains supplementary material available at 10.1186/s40643-025-00839-y.

## Introduction

Carboxylates are compounds widely used in industrial sectors producing polymers, adhesives and solvents. Carboxylates include volatile fatty acids (VFAs), ranging from two to six carbon atoms, whose market is expected to grow in the coming years. Acetic, propionic and butyric acids are the most industrially produced. In the case of acetic acid and propionic acid, an annual growth rate of 7.5% and 3.3%, respectively, is projected for the period 2024 to 2030 (Grand View Research 2023; Straits Research 2024). Replacing the use of fossil oils to obtain VFAs is possible because they can also be produced by using biotechnological means via open-mixed cultures. This biotechnological approach is regarded as a powerful production strategy to decrease society’s dependency on petroderivatives.

VFAs production via anaerobic fermentation is gaining interest as sustainability trends drive business transformation. The so-called carboxylate platform aims to convert waste into bioproducts by harnessing the activity of diverse microbial consortium of anaerobes in which the methanogens are inhibited. Anaerobic digestion has traditionally been used to produce bioenergy in the form of biogas with active methanogenesis (Kumar et al. 2021). Anaerobic digestion consists of four main steps: hydrolysis (carbohydrates, proteins, lipids are hydrolysed by bacteria), acidogenesis (conversion of simple monomers from the hydrolytic step into VFAs, CO_2_, H_2_, ammonia, and hydrogen sulphide by fermentative bacteria), acetogenesis (oxidation of acidogenesis products to obtain acetate, CO_2_ and H_2_ to be consumed by methanogenic archaea for methane production or by acidogenic bacteria to produce VFAs), and methanogenesis (production of CH_4_ by methanogenic archaea) (Magdalena et al. 2019). But in the last decade, this bioprocess has been the focus of intensive research to produce VFAs (Zhou et al. 2018). When methanogens´ activity is arrested, VFAs are accumulated in the culture broth instead of being consumed for biogas production purposes. This shortened bioprocess (comprising only the hydrolysis and acidogenesis steps) can be attained by optimising the operational parameters of the fermentative stage. Mild temperatures (25–35 ºC) (Greses et al. 2020, 2021), slightly acidic pH (5–6) (den Boer et al. 2020; Greses et al. 2022a), high organic load rates (OLRs) (Calero et al. 2018; Pereira et al. 2022) and low hydraulic retention times (HRTs) (Ri et al. 2019; Sun et al. 2021) have been proved to be good strategies to promote acidogenic fermentation (AF).

The AF of organic residues to produce carboxylates has been investigated in different reactor configurations including continuous stirred tank reactors (CSTR) (Greses et al. 2022b), sequencing batch reactors (SBR) (Lagoa-Costa et al. 2020), fluidised bed reactors (FBRs) (Borja et al. 1995), packed bed reactors (PBRs) (Fuess et al. 2016) or up-flow sludge bed reactors (UASB) (Calero et al. 2018). By far, CSTRs have been mostly used, while some other configurations could bring important key features for the production of these VFAs. This is the case of UASB reactors, which have a simple design and low maintenance and operating costs. These reactors have been shown to be able to reach high treatment efficiencies even under challenging operation conditions, such as high OLRs and low HRT (Daud et al. 2018; Narayanan and Narayan 2019). This type of reactor usually works with granular sludge, which provides a compact and high density microbial structure that enables an easier biomass settlement (Chen et al. 2021). This is a crucial feature when it comes to the field of VFAs production, since their recovery out of the cultivation broth is highly dependent on the amount of solids and other impurities present in the effluents. Indeed, the economic feasibility of this technology has been claimed to be highly dependent on the recovery technologies (Magdalena et al. 2019). Because of that, UASB, a configuration that could be operated at really low biomass/solids concentration in the effluent, could be a key enabler of this technology by which recovery cost might be considerably decreased.

In general, high carbohydrate content wastes are the most suitable feedstocks for AF, as they mediate high bioconversion efficiencies (Greses et al. 2021, 2022b). The composition of molasses, which is rich in carbohydrates (up to 50% (Carioca and Leal 2011), mainly sucrose (Palmonari et al. 2020), makes this by-product an easily degradable substrate suitable for AF processes. In this way, molasses could be a promising substrate to expand the portfolio of feedstocks contributing to the carboxylate platform. Molasses is a by-product of the sugar industry, consisting of a thick syrup containing the non-extractable sugars from sugar cane or sugar beet. It is known to be a cheap substrate (Ajala et al. 2021) and is usually used as a raw material for the production of amino acids, citric acid, lactic acid by yeast and fermentation industries (EBSSP 2017), and also for the animal feed industry. Some studies on anaerobic digestion or dark fermentation using molasses have been reported in the literature, but these mainly focus on the production of biogas (Ribeiro et al. 2024a) or hydrogen along with other metabolites such as ethanol and lactic acid (Ribeiro et al. 2024b).

The production of VFAs using UASB reactor configuration has been scarcely studied, while the use of this configuration has been mostly employed for biogas production (Le et al. 2024). Nevertheless, this configuration, which allows compact and cheaper fermenters designs should not be disregarded for VFAs production when the feedstock is a wastewater-type. Because of that, the objective of this research was to study the AF process by using the UASB reactor configuration, focusing on the effects of different inoculum sources (and thereby composed by different microbial communities) and OLR on VFAs production and process efficiency using a carbohydrate-rich waste (sugar cane molasses). For this purpose, preliminary biochemical carboxylate potential (BCP) assays were carried out to determine the optimal temperature and the most suitable inoculum. Once the general trends in BCPs were determined, the robustness of the bioprocess was assessed in continuous feeding mode by operating UASB reactors.

## Materials and methods

### Feedstock and inoculum characterization

Sugar cane molasses, supplied by Compañía de Melazas S.A (Madrid, Spain), was used as feedstock for the AF process. Molasses was selected as feedstock due to its high carbohydrates content, and readily degradable organic share, which facilitates the steps of hydrolysis and acidogenesis. The chemical characterization (total chemical oxygen demand (TCOD), soluble chemical oxygen demand (SCOD), total solids (TS), volatile solids (VS), pH and content in carbohydrates, lipids, proteins and ashes) of this waste is shown in Table 1.

**Table 1:** Chemical characterization of the sugar cane molasses used as feedstock

	Sugar cane molasses	
TCOD (g·L^− 1^)	1120	± 24
SCOD (%)	96.5	± 3.3
TS (g·L^− 1^)	1030	± 6
VS (%) ª	85.7	± 0.5
pH	7.0	± 0.1
Carbohydrates (wt%) ^a^	57.3	± 4.5
Proteins (wt%) ^a^	7.6	± 0.1
Lipids (wt%) ^a^	20.8	± 1.4
Ash (wt%) ^a^	14.3	± 0.1

^a^Calculated in a dry matter basis

Two different anaerobic granular sludge were selected as inoculum for this investigation. One was collected in a brewery company (Guadalajara, Spain) (brewery granular sludge, BGS) and was characterized by a content of 46.6 ± 0.6 g TS L^-1^, 39.5 ± 0.4 g VS L^-1^, 0.5 ± 0.1 g N-NH_4_^+^· L^-1^ and pH 7.5 ± 0.1. The second sludge (paper plant granular sludge, PGS) was collected from a paper plant company (Palencia, Spain) and was characterized by a content of 46.3 ± 0.6 g TS L^-1^, 39.1 ± 0.4 g VS L^-1^, 0.5 ± 0.1 g N-NH_4_^+^· L^-1^ and pH 7.0 ± 0.1. Both inocula were similar in terms of TS, VS, ammonium nitrogen content (N-NH_4_^+^) and pH.

### Biochemical carboxylate potential test

First, BCPs were carried out to determine the optimal temperature and the most suitable granular inoculum for the AF of molasses by following the BCP test procedure described by Magdalena and González-Fernández 2020. The AF was performed in 120 mL batch reactors with a working volume of 70 mL. The initial substrate-to-inoculum ratio was set at 3 g COD_substrate_/g VS_inoculum_ in order to inhibit methanogenic activity (González-Fernández and García-Encina 2009). To evaluate the endogenous VFAs production of the inocula, blank tests were run using water instead of substrate. Initial pH in the BCPs was adjusted to 7 using a NaOH solution (5 M) to avoid sudden acidification of the media (Zhou et al. 2018). No pH control was conducted after the start-up of BCPs. The experiment was carried out at 25 ºC, 35 ºC and 55 ºC in orbital shakers (AG CH-4103 Bottmingen, Infors). Twice a week, the VFAs concentration was measured by extracting 0.5 mL of sample from the reactors, and subsequently filtered by using a 0.2 µl filter. BCP tests were run until the production of VFAs was stable (approximately 16–17 days), and the optimal temperature will be selected among the experiments with a better VFA production and bioconversion. All assays, including blank tests, were run in triplicate.

### UASB reactors description and operation

Once the optimum temperature was selected, semi-continuous fed AF of molasses was performed in UASB reactors. In this case, two UASB reactors were used. The first UASB had a working volume of 4.2 L (height of 21 cm and an internal diameter of 10.6 cm) and was inoculated with the PGS sludge. The second one was characterised by a working volume of 5 L (height of 23.1 cm and an internal diameter of 10.6 cm) and was inoculated with BGS sludge. Based on previous results (Lago et al. 2023), both reactors were operated at an HRT of 10 days and an OLR of 3 g_COD_·L^-1^·d^-1^. The pH was adjusted in the range of 5.5-6 with NaOH (5 M) or H_2_SO_4_ (5 M) solutions, when necessary. This pH range was selected based on the fact that this range has been reported to be optimum for VFAs production (Greses et al. 2022a). To ensure a good mixing in the reactors, the supernatant was recirculated every 2 h using a peristaltic pump (L100-1 S-2, Longer precision pump Co.) at a flow rate of 50 mL·min^-1^. UASB reactors were operated until the stationary phase was reached. Subsequently, the UASB reactor with the highest efficiency underwent a stepwise increase in OLR, starting from 3 g_COD_·L^-1^·d^-1^ and progressively rising to 4.5 g_COD_·L^-1^·d^-1^ and to 6 g_COD_·L^-1^·d^-1^, achieving stability on days 110 and 189, respectively. The performance of the UASB reactors was monitored by analyzing the effluents twice a week for TCOD, SCOD, TS, VS, pH, and VFAs. Stability was defined as the point at which these parameters reached stationary values. Additionally, biogas production and composition were regularly monitored. Samples of the microbial systems were collected at the start of operation and during the steady state periods and stored at -80 ºC to analyse the microbial communities.

### Analytical methods

TCOD and SCOD were determined using a Spectroquant^®^ COD cell test (MerckMillipore), after filtering the effluent sample through a 0.45 μm filter for SCOD. N-NH_4_^+^ content was quantified using a Spectroquant^®^ ammonium test (MerckMillipore). TS, VS and ash content of the feedstock, inocula and effluents were measured according to standard methods (APHA/AWWA/WEF 2017). pH was measured daily using a pH-meter (Hach-Lange SENSION + pH31). Chemical characterization of the feedstock was conducted with different methods. The carbohydrate content was measured using the sulphuric-phenol method (Dubois et al. 1951). Protein content was measured by determining the total nitrogen content by Kjeldahl method (Owusu-Apenten 2002) and applying a conversion factor of 6.25 (Moore et al. 2010). Lipid content was calculated by subtracting the carbohydrates, proteins and ash content from total solids. Biogas composition (hydrogen, carbon dioxide and methane) was determined by gas chromatography coupled with a thermal conductivity detector (Clarus 580 GC, PerkinElmer) equipped with a HSN6-60/80 Sulfinert P packed column (7 × 1/8´´ O.D.) and a MS13 × 4-09SF2 40/60P packed column (9 × 1/8´´ O.D.) (PerkinElmer), using the same conditions as described by Magdalena et al. 2020. VFAs, lactic acid and ethanol concentrations were measured by liquid chromatography using an Agilent 1260 HPLC equipped with a refractive index detector, a pre-column (Cation H Refill Cartridge Microguard column, Biorad) and an ion exclusion column (Aminex HPX-87 H 300 × 7.8 mm I.D., Biorad) under the conditions used by Llamas et al. 2021.

Once the UASB reactors reached the steady state (TCOD, SCOD, TS, VS and VFA concentrations were stable), the efficiency of the AF was assessed by calculating the bioconversion efficiency, hydrolysis degree (SCOD/TCOD), acidification degree (COD_acidified_) and COD removal by using the following equations (Greses et al. 2020, 2021) (Eqs. (1)-(4):

1 $$\text{\%\:Bioconversion\:efficiency}\text{\:=\:}\frac{{\text{COD}}_{{\text{VFAs}}_{\text{effluent}}}}{{\text{TCOD}}_{\text{influent}}} {\cdot } \text{\:\:\:100}$$

2 $$\:\text{\%\:}\text{SCOD/TCOD}\text{\:}\text{=}\frac{{\text{SCOD}}_{\text{effluent}}}{{\text{TCOD}}_{\text{effluent}}}\text{\:}{\cdot}\text{\:}\text{100}$$

3 $$\:\text{\%\:}{\text{COD}}_{\text{acidified}}\text{\:=}\frac{{\text{COD}}_{{\text{VFAs}}_{\text{effluent}}}}{{\text{SCOD}}_{\text{effluent}}} {\cdot} \text{\:\:100}$$

4 $$\:\text{\%\:}{\text{COD}}_{\text{removal}}\text{\:}\text{=}\frac{{\text{TCOD}}_{\text{influent}}-{\text{TCOD}}_{\text{effluent}}}{{\text{TCOD}}_{\text{influent}}}\text{\:} {\cdot}\text{\:}\text{100}$$

where COD_VFAseffluent_ represented the total concentration of acetic acid (HAc), propionic acid (HPro), isobutyric acid (isoHBu), butyric acid (HBu), isovaleric acid (isoHVal), valeric acid (HVal) and caproic acid (HCa) in the UASB reactor effluents measured as g COD L^-1^. The COD equivalents of the VFAs according to oxidation reaction stoichiometry were 1.07 for HAc, 1.51 for HPro, 1.82 for HBu and isoHBu, 2.04 for HVal and isoHVal and 2.20 for HCa in gCOD gVFA^-1^ (Greses et al. 2022c). TCOD_influent_ was the concentration of TCOD of the feedstock fed into the reactors (g COD L^-1^), SCOD_effluent_ was the soluble fraction of COD analysed in the reactors’ effluents (g COD L^-1^) and TCOD_effluent_ represented the TCOD concentration determined in the semicontinuous reactors’ effluents (g COD L^-1^).

### Microbial community identification procedure

To correlate the chemical output with the performance of the biological system, microbial communities were analysed in the steady phase of the experiments. For that purpose, DNA extraction was performed using SPIN kit for Soil (MP Biomedicals, LCC) from 1 mL of sample according to the manufacturer´s procedure. Quantity and quality of the extracted DNA were measured using a Nanodrop spectrophotometer (SPECTROstar Omega e BMG Labtech, DE). DNA samples were sequenced by FISABIO (Valencia, Spain) on a MiSeq Sequencer (Illumina) using the primers 341 F (F-CCTACGGGNGGCWGCAG) and 805R (R-GACTACHVGGGTATCTAATCC) to specifically target the hyper-variable regions V3 and V4 of the 16 S rRNA gene for both bacteria and *Archaea*. The obtained sequences were processed to identify the microorganisms present in the samples as described by Greses et al. (Greses et al. 2021). Biodiversity indexes (observed operational taxonomic units (OTUs) at 97% identity and Shannon index) were calculated using QIIME 1.9.1 software package (Caporaso et al. 2010).

## Results and discussion

### VFAs production in BCP tests: the effect of fermentation temperature and inoculum source

The results obtained in the BCP tests are presented in Table 2. As can be observed, the highest bioconversion was obtained at 25 ºC for both inoculum sources. The highest values of bioconversions achieved were 40.4 ± 0.2% for BCP-BGS-25 ºC and 27.3 ± 0.3% for BCP-PGS-25 ºC. Further information on VFAs production was provided in the Supporting information.

**Table 2:** Composition of BCP test effluents determined in the steady state of the process

	BCP-PGS-25	BCP-PGS-35	BCP-PGS-55	BCP-BGS-25	BCP-BGS-35	BCP-BGS-55
pH	4.0 ± 0.1	4.1 ± 0.1	4.8 ± 0.1	4.2 ± 0.1	3.9 ± 0.1	4.7 ± 0.1
TCOD (g·L ^− 1^)	55.5 ± 0.1	91.8 ± 5.7	93.1 ± 3.5	77.5 ± 4.2	77.2 ± 5.7	75.5 ± 0.2
SCOD/TCOD (%)	81.7 ± 4.7	45.2 ± 8.4	56.2 ± 3.2	62.7 ± 1.1	62.2 ± 8.4	70.9 ± 3.4
VFAs total (g·L^− 1^)	8.9 ± 0.1	7.5 ± 0.2	7.1 ± 0.4	13.8 ± 0.1	5.9 ± 0.2	5.9 ± 0.1
HAc (%) ^a^	44.5 ± 1.1	95.1 ± 1.0	17.8 ± 1.3	62.0 ± 0.2	48.7 ± 1.0	14.2 ± 0.2
HPro (%) ^a^	6.7 ± 0.3	4.2 ± 0.9	< LD ^b^	2.2 ± 0.0	5.1 ± 0.9	0.8 ± 0.7
HBu (%) ^a^	48.7 ± 0.8	4.9 ± 0.1	81.3 ± 1.9	37.9 ± 0.4	46.2 ± 0.1	84.7 ± 0.4
Lactic acid (g·L^− 1^)	14.1 ± 0.3	15.1 ± 0.2	5.6 ± 0.1	6.6 ± 0.1	16.9 ± 0.2	4.0 ± 0.1
Bioconversion (%)	27.3 ± 0.3	16.8 ± 0.4	15.6 ± 0.8	40.4 ± 0.2	18.0 ± 0.4	12.9 ± 0.3
Bioconversion (+ lactic acid) (%)	60.2 ± 0.6	51.9 ± 0.5	28.4 ± 0.8	55.9 ± 0.6	56.9 ± 0.5	27.7 ± 0.2
COD_acidified_ (%)	28.2 ± 0.3	27.5 ± 0.4	24.4 ± 0.3	39.2 ± 0.2	17.3 ± 0.4	18.8 ± 0.2

^a^ VFA/TotalVFAs in g·L^-1^· 100

^b^ Lower than Limit of Detection

Bioconversion efficiencies were significantly lower when the AF temperature was higher than 25 ºC, being 18.0 ± 0.2% and 12.9 ± 0.3% for BGS at 35 ºC and at 55 ºC, respectively, and 16.8 ± 0.4% and 15.6 ± 0.8% for PGS at 35 ºC and at 55 ºC, respectively. Similar to the bioconversion efficiencies, the acidification efficiencies also decreased concomitantly with an increase in AF temperature. As a matter of fact, although AF at 25 ºC resulted in higher bioconversions than those recorded at 35 ºC and 55 ºC, all values were lower than the ones reported in the literature for batch reactors (Greses et al. 2022c; Lago et al. 2023), evidencing an inhibition of the acidogenic step. This phenomenon could be explained by the low pH values reached in the BCPs (< 5), which hindered the acidogenic activity, as evidenced by the accumulation of intermediate metabolites (such as lactic acid) due to the change in the metabolic pathways that provoked the accumulation of these primary metabolites instead their conversion to VFAs (Greses et al. 2022a). Indeed, there was a high lactic acid accumulation at 35 ºC for BGS (16.9 ± 0.7 g·L^-1^) and at 25 ºC and 35 ºC for PGS (14.1 ± 0.3 g·L^-1^ and 15.1 ± 0.2 g·L^-1^, respectively). This lactic acid accumulation was the responsible of the low bioconversion to VFAs, as it is a primary metabolite that should be converted to VFAs in an optimal AF (Wainaina et al. 2019a). In BCP-BGS-25, the accumulation of lactic acid did not reach such high concentrations, thereby explaining the higher bioconversion to VFAs.

Regarding the effect of temperature and sludge source on the VFA profile, both HAc and HBu production were enhanced at low temperature (Table 2), being the main carboxylic acids produced at 25 ºC. However, their proportion was different depending on the sludge microbial community. In BCP-BGS-25, HAc was the prevailing carboxylate with a 62.0 ± 0.2% of the total VFA, followed by HBu with a 37.9 ± 0.4%. In contrast, HBu was the main VFA in BCP-PGS-25, accounting for 44.5% of the total carboxylate pool. Increasing the fermentation temperature to 35 ºC also resulted in a change in the distribution profile for each microbial source. In BCP-BGS-35, HAc was still predominant but its proportion decreased to 48.7 ± 0.1% when compared to 25 ºC, while HBu increased to 46.2 ± 0.2% of the total carboxylates. In BCP-PGS-35, HAc became the main acid, being 95.1 ± 1.0% of the total VFAs. BCPs results were in line with the expected metabolic pathways commonly described at those pHs (Zheng et al. 2015; Feng et al. 2018). Heterolactic fermentation, with HAc as a by-product, is commonly attained at a pH of 4.0 (Zhou et al. 2018; Feng et al. 2018). This metabolic pathway seemed to be followed in BCP-PGS-35 (production of lactic acid, HAc along with CO_2_ and absence of H_2_ (Castillo Martinez et al. 2013), and partially in BCP-BGS-25, BCP-BGS-35 and BCP-PGS-25. In the experiments conducted in the thermophilic range (55 ºC), the presence of HBu became more evident showing a metabolic combination of this heterolactic fermentation with a butyric-type fermentation due to the pH increase (Feng et al. 2018). This behaviour was observed in all BCPs at 55 ºC regardless of the granular sludge used as inoculum, revealing the sharp effect of high temperature on microbial performance.

These results demonstrated the feasibility of producing VFAs via AF using granular sludge. Mild temperatures (25 ºC) mediated a higher bioconversion of the substrate to VFAs than the other temperatures. pH was evidenced to be a key factor in the AF process. Acidic pH (< 5) led the process to the accumulation of lactic acid, hindering the production of VFAs, presumably by changes in metabolic pathways due to the prevalence of acid resistant and lactic acid producing bacteria (Greses et al. 2022a). In this way, this parameter needs to be controlled to promote VFAs accumulation (pH = 5.5-6) (Greses et al. 2022a). These preliminary results attained in BCP experiments represented general tendencies. Because of that semicontinuous operated reactors were run to verify the results of BCPs. For this purpose, both inocula were used in UASB reactors at 25 ºC to identify which microbial community was more suitable for VFAs production.

### Effect of inoculum source in semicontinuosly operated UASB reactors: chemical and biological performance

**Fig. 1:**
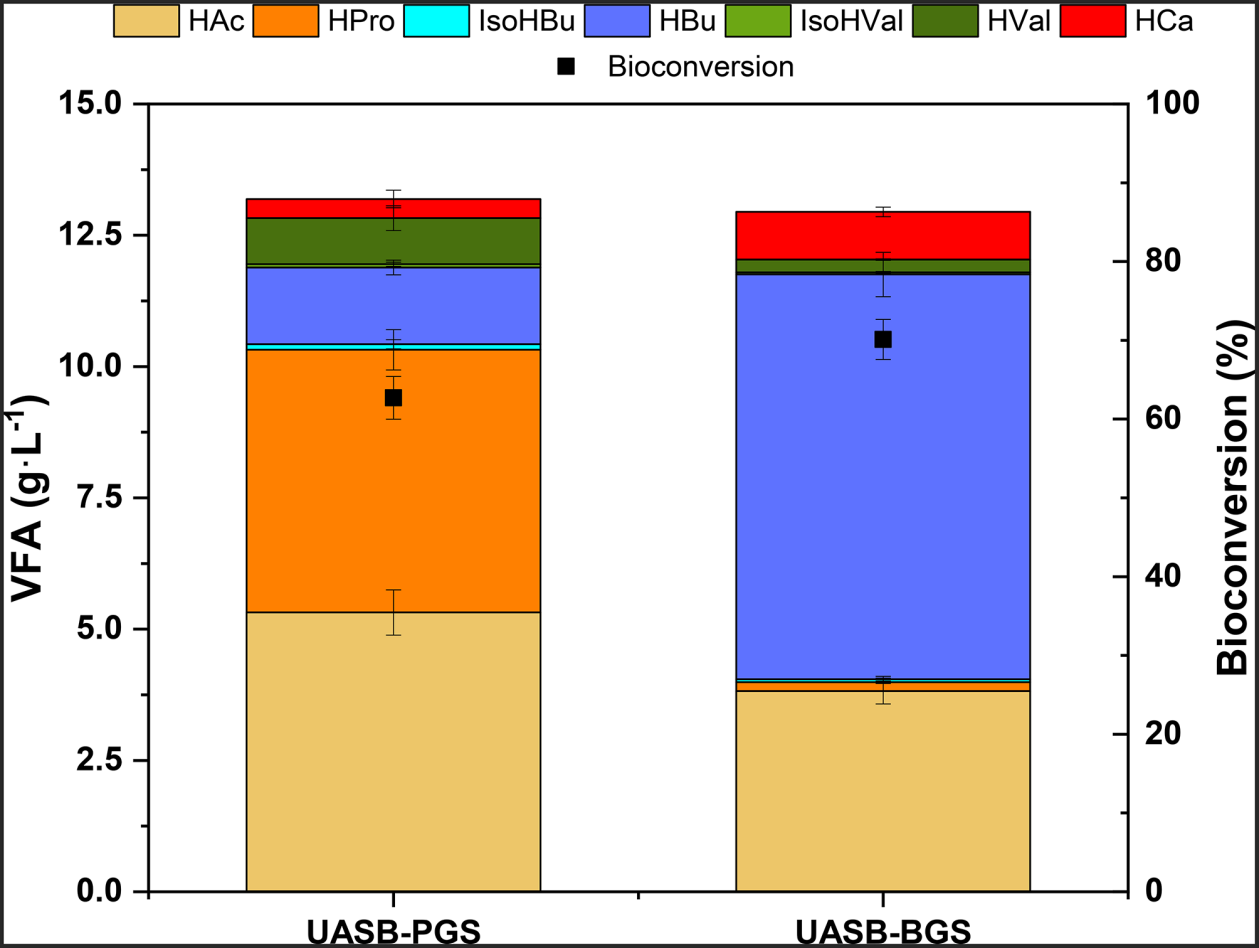
Effect of the type of inoculum for VFAs production, distribution and bioconversions in the AF of sugar cane molasses in UASB reactors

The process was scaled up to a semicontinuous operated UASB reactor configuration. Although the use of PGS in BCPs was not as favourable as with BGS, this inoculum was also tested in UASB reactors, where pH can be easily controlled in the optimal range for VFAs production (5.5-6). In this way, lactic acid accumulation could be prevented via pH adjustment, thus promoting VFAs accumulation. The results obtained in the UASB reactors using BGS and PGS inocula are presented in Fig. 1; Table 3.

**Table 3:** Composition of UASB reactors effluents in the steady state using PGS and BGS as inocula

	UASB-PGS		UASB-BGS	
pH	5.8	± 0.3	5.8	± 0.2
TCOD (g·L^− 1^)	30.5	± 1.8	27.4	± 1.2
SCOD/TCOD (%)	89.1	± 4.8	93.4	± 1.7
TS (g·L^− 1^)	25.7	± 1.2	23.6	± 1.6
VS (g·L^− 1^)	13.9	± 1.4	13.5	± 0.8
VFAs total (g·L^− 1^)	13.2	± 0.5	12.9	± 0.5
Lactic acid (g·L^− 1^)	0.1	± 0.0	0.5	± 0.4
Ethanol (g·L^− 1^)	0.1	± 0.0	<	LD^b^
Bioconversion (%)	62.7	± 2.7	70.1	± 2.5
COD_acidified_ (%)	67.7	± 4.1	82.3	± 3.7
COD removal (%)	3.1	± 1.1	6.6	± 1.5
CH_4_ yield (mL·g_COD_^−1^·d^− 1^)	10.9	± 4.3	0.9	± 0.8

^a^ VFA/TotalVFAs · 100

^b^ Lower than Limit of Detection

The VFAs production in UASB-PGS was 13.2 ± 0.5 g·L^-1^, accounting for a bioconversion efficiency of 62.7 ± 2.7%. This result outperforms previous studies using different reactor configurations (CSTR or Anaerobic sequencing batch reactor (AnSBR) with similar substrates, such as sugar industry wastewater (Alkaya and Demirer 2011), carbohydrate-rich residues, (Greses et al. 2021) or beet molasses (Lago et al. 2023), reaching 52.9, 46.3 and 55.5% bioconversion respectively. Compared to the BCPs, the bioconversion efficiency achieved in semicontinuously operated UASB was much better than those achieved in BCP tests using PGS as inoculum (62.7 ± 2.7% in UASB-PGS vs. 27.3 ± 0.3% in BCP-PGS-25). In addition, the hydrolysis percentage (from 62.7 ± 1.1% in BCP-PGS-25 to 89.1 ± 4.8% in the UASB-PGS) and acidification efficiency (from 28.8 ± 0.3% in BCP-PGS-25 to 67.7 ± 4.1% in the UASB-PGS) were also improved. This enhancement of hydrolysis rate, acidification and the bioconversion efficiencies could be explained by the pH control implemented in semicontinuous operation. In UASB-PGS, the pH (5.8 ± 0.3) was in the optimal range for VFAs accumulation (5.5-6) (Jiang et al. 2013). Maintaining the pH at optimal levels led to a decrease in lactic acid accumulation in the reactor, improving VFAs accumulation and AF bioconversion efficiency.

A similar trend was observed in the experiments using BGS as inoculum. The pH control in the semicontinuously operated reactor also mediated a bioconversion efficiency improvement from 40.4 ± 0.2% in BCP-BGS-25 to 70.1 ± 2.5% in the UASB-BGS. This was also reflected in the negligible lactic acid accumulation in this reactor (0.5 ± 0.4 g·L^-1^) compared to BCP-BGS-25 (6.6 ± 0.1 g·L^-1^, Table 2). In accordance to the higher bioconversion, the pH control also enabled an enhanced hydrolysis and acidogenesis rates (SCOD/TCOD = 93.4 ± 1.7%; COD_acidified_ = 82.3 ± 3.7%) since the lactic acid produced in the fermentation was transformed into VFAs.

With respect to the solids concentration in the effluents, both reactors achieved similar levels of total solids (TS) and volatile solids (VS): 25.7 ± 1.2 g TS/L and 13.9 ± 1.4 g VS/L in UASB-PGS, and 23.6 ± 1.6 g TS/L and 13.5 ± 0.8 g VS/L in UASB-BGS. These concentrations exceeded the reduction of solids in the effluent stream obtained when using CSTRs (Lago et al. 2023). 0.51 g VFAs/g TS in UASB-PGS and a 0.55 g VFAs/g TS in UASB-BGS can be calculated from the chemical characterisation of the effluents obtained in the UASBs while using a CSTR with a similar substrate, this ratio decreased to 0.39 g VFAs/g TS (Lago et al. 2023). This ratio indicated that more VFAs were produced per gram of TS in the effluent stream, highlighting one of the more useful characteristics of UASB reactors, the obtaining of a low solids content effluent. This is an important feature as the downstream processing of VFAs depends on VFAs extraction/purification efficiency.

With the calculated COD removals, it could be confirmed that there was no VFAs consumption to produce biogas, as can be seen in Table 3 (COD removal of 3.1 ± 1.1% and a production of methane of 10.9 ± 4.3 mL·g_COD_-1·d^-1^ for UASB-PGS and 6.6 ± 1.5% and 0.9 ± 0.8 mL·g_COD_^-1·^d^-1^ for UASB-BGS). These low values of COD removal and methane production demonstrated that the implemented operational conditions were optimal for the production of VFAs, as the archaea community was hampered and no methane was produced.

It is important to highlight that both UASB reactors exhibited a very similar total VFA production (Table 3), solids concentration and COD removal, but UASB-BGS resulted in higher bioconversion efficiency (70.1 ± 2.5%) than UASB-PGS (62.7 ± 2.7%). This difference in bioconversion efficiencies was attributed to the VFAs profile dissimilarity. As it can be seen in Fig. 1, the main VFAs produced in UASB-PGS were HAc (40.3 ± 2.0%) and HPro (38.0 ± 4.0%), while HBu (59.5 ± 2.4%) and HAc (29.5 ± 1.7%) were the prevailing VFAs in the UASB-BGS followed by HCa (7.0 ± 0.6%). The presence of longer carbon chain VFAs (> C4) in UASB-BGS contributed to a greater extent to the SCOD of the effluent and thereby, a higher bioconversion efficiency was determined in this reactor.

Comparing the bioconversion and VFAs production in the BCP tests and the experiments carried out in the UASB reactors, it can be concluded that pH was a key factor in AF performance targeting VFAs. Whereas BCPs at 25 ºC and pH 3.9–4.2 resulted in the accumulation of lactic acid, a controlled pH in the range of 5.5-6 in UASBs (25 ºC) provoked the disappearance of lactic acid in favour of VFAs production. This effect was also addressed in the AF of vegetable wastes in CSTR (Greses et al. 2022a).

Regarding the VFAs distribution profiles, the results obtained from UASB-BGS were in accordance with previous data attained for carbohydrate-rich residues, such as beet molasses (Lago et al. 2023) or agroindustrial wastes (Greses et al. 2020). In this reactor, even-chain carboxylic acids like HAc and HBu prevailed over odd-chain VFAs, which has been commonly detected in recent studies dealing with molasses valorisation into VFAs using different reactor configurations, such as SBR or CSTRs (Lago et al. 2023). Nevertheless, UASB-BGS resulted in a higher bioconversion than that attained in CSTR (55.5 ± 1.5% (Lago et al. 2023) vs. 70.1 ± 2.5%), demonstrating the relevance of biomass-retaining configurations on AF performance.

Conversely, the VFAs profile attained in UASB-PGS was not very common using this type of substrate and conditions. Previous studies concluded that lactic acid plays a key role in propionic-type fermentation in presence of specific syntrophic bacteria, such as *Propionibacterium* (Valdez-Vazquez et al. 2005; Chen et al. 2013), therefore it is important to further understand the different evolution of VFAs profile between the UASBs.

**Fig. 2:**
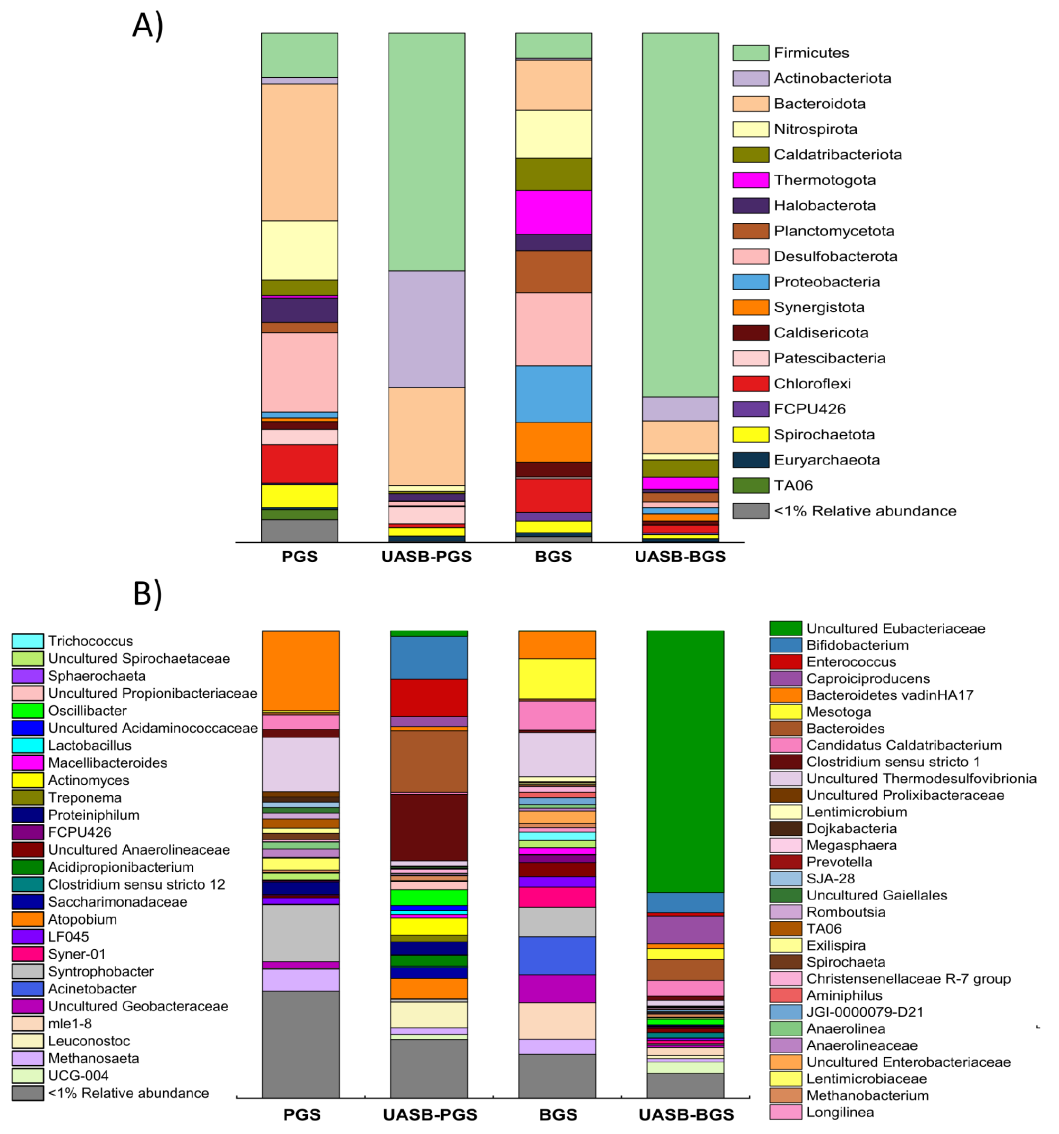
Relative abundance of bacterial and archaeal OTUs at phylum (**A**) and genera (**B**) level in PGS and BGS inoculum, in UASB-PGS and in UASB-BGS

For this purpose, a microbial community analysis of both inocula and both microbiomes developed in the UASB reactors was performed. The relative abundance of bacterial and archaeal OTUs at phylum and genus levels was investigated (Fig. 2).

As it can be seen in Fig. 2, the microbial profile at phylum level changed considerably from the inocula to those detected in both UASB reactors at the steady state. Similar phyla were identified in the inocula (PGS and BGS), including Firmicutes, Bacteroidota, Desulfobacterota, Nistrospirota, Chloroflexi or Synergistota. These microorganisms are commonly found in anaerobic systems for biogas production using granular sludge (Callejas et al. 2022). However, some differences were observed between the inocula that were mainly attributed to the development of substrate-specific bacteria. For instance, PGS showed a higher relative abundance of Bacteroidota (26.9%) than BGS (9.9%), mainly composed of *Bacteroidetes vadin HA17* (Fig. 2b). These bacteria have been previously identified in papermill wastewater effluents due to the presence of recalcitrant compounds, such a lignin-derivatives (Doloman et al. 2024). In contrast, the presence of organic compounds (namely soluble starch and ethanol) in brewery wastewater promoted the development of Thermotogota (7.66%, namely *Mesotoga*), Proteobacteria (11.10%, *Enterobacteriaceae*), Planctinomycetota (8.24%, *mle1-8*) and Desulfobacterota (15.6%, *Geobacteriaceae*) (Doloman et al. 2024).

Compared to PGS inoculum, the microbial community established when stability was reached in UASB-PGS changed substantially. The microbiome analysed in UASB-PGS prevailed in Firmicutes (46.7%), Actinobacteriota (22.9%) and Bacteroidota (19.3%). It should be highlighted that these phyla were consistent with those recognized as butyrate-producing bacteria (Zhu et al. 2021). The reduction of Chloroflexi, Desulfobacterota and Nitrospirota phyla was due to the conditions imposed on the reactors, such as acidic pH and feedstock composition (rich in carbohydrates). This feature was also reported for other type of reactors with similar operational conditions (Greses et al. 2021).

The high relative abundance of Firmicutes was strongly associated with the production of VFAs from carbohydrates-rich feedstocks (Jaenicke et al. 2011). Firmicutes phylum in UASB-PGS was mainly composed by *Clostridium sensu stricto 1* genus (14.3%) and *Enteroccoccus* (10.3%). The abundance of *Clostridium sensu stricto 1* is related to the production of acetate and butyrate (Łukajtis et al. 2018), which were the products determined in the AF broth in UASB-PGS while *Enteroccoccus* genus abundance is related to the production of lactic acid (Candry et al. 2020). In addition, *Oscillibacter* genus (3.5%) could explain the presence of valeric acid in the media (Iino et al. 2007).

The production of HPro and HAc was related to the abundance of Bacteroidota from the degradation of lactic acid (Sarkar et al. 2016). Among these microorganisms, *Bacteroides* genus have been reported to be HAc and HPro producers from lactate (Zhao et al. 2008; Sarkar et al. 2016), explaining the high production of these carboxylates and the absence of lactic acid in the reactor.

Actinobacteria phylum bacteria are associated with the degradation of carbohydrates to produce lactic acid, HAc and HPro (Greses et al. 2020; Lim et al. 2020). This phylum was mainly composed by *Bifidobacteium* (6.5%), *Actinomyces* (4.3%) and *Atopobium* (2.8%) genera, which are responsible for lactic acid production and HAc (Hamilton and Ellwood 1983; Gulhane et al. 2017; Zhou et al. 2018). Although the presence of lactic acid was negligible in UASB-PGS reactor, the presence of these microorganisms was justified as lactic acid was presumably consumed to produce long chain-VFAs.

Similarly to UASB-PGS, the fermentation conditions imposed in UASB-BGS led to a drastic microbial profile change. Firmicutes (71.4%), Bacteroidota (6.4%) and Actinobacterota (4.6%) were the prevailing phyla when this UASB reached stability. The increase in Firmicutes abundance coincided with the decrease of Bacteroidota and Actinobacteriota abundance. These changes in microbial communities at phylum level likely contributed to the differences in VFAs profiles (Fig. 2). This is confirmed by the presence of uncultured *Eubacteriaceae* family microorganisms (56.0%), known producers of medium-chain fatty acids and HBu (Greses et al. 2023). The presence of *Caproiciproducens* genus, chain elongators microorganisms (6.0%), justified the relative high presence of HCa (7.0 ± 0.6%), produced via reverse β-oxidation (Stamatopoulou et al. 2020).

The lower amount of Bacteroidota microorganisms in the UASB-BGS microbiome could explain the differences in HPro production between UASB-PGS (38.0 ± 4.0%) and UASB-BGS-3 (1.3 ± 0.2%). In accordance with the decrease of Bacteroidota phylum abundance, the reduction of Actinobacteria abundance in UASB-BGS microbial culture, confirmed the changes in VFA profile compared with UASB-PGS.

In conclusion, the use of pH control in UASB reactors allowed diminishing the accumulation of lactic acid, which promoted the accumulation of VFAs as well as the bioconversion efficiency. In addition, working with this type of reactor also brought higher bioconversion efficiencies compared to other reactor configurations, such as CSTR and AnSBR, opening the possibility to work at lower HRT and higher OLR. On the other hand, the differences observed in VFA profiles can be attributed to the development of different microbial communities (UASB-PGS and UASB-BGS) due to the different inoculum sources employed.

### Identifying the OLR threshold that decrease the bioconversion efficiency: chemical and microbial performance

PGS inoculum led to a VFAs profile enriched in HAc and HPro, whereas for BGS, the prevailing carboxylate was HBu. Larger chain carboxylic acids have a higher market price (Sukphun et al. 2021), thereby being more interesting from an economic point of view. In addition, having a less diverse carboxylate production profile could help reducing the complexity and operational costs for further VFAs separation processes. Using BGS allowed to fulfill these premises, thus, this microbiome was selected to identify the OLR threshold that would hinder an optimum bioconversion efficiency. The OLR was first increased from 3 (UASB-BGS-3) to 4.5 g COD·L^-1^·d ^-1^ (UASB-BGS-4.5), and then further increased to 6 g COD·L^-1^·d ^-1^ (UASB-BGS-6).

**Table 4:** UASB reactor effluent composition using BGS stepwise OLR increase from 3 g COD·L^-1^·d^-1^ to 6 g COD·L^-1^·d^-1^

	UASB-BGS-3	UASB-BGS-4.5	UASB-BGS-6
pH	5.8 ± 0.2	5.7 ± 0.1	5.7 ± 0.1
TCOD (g·L^− 1^)	27.4 ± 1.2	43.3 ± 0.9	49.9 ± 1.3
SCOD/TCOD (%)	93.4 ± 1.7	90.1 ± 2.9	89.3 ± 2.9
TS (g·L^− 1^)	23.6 ± 1.	33.8 ± 1.4	45.0 ± 1.4
VS (g·L^− 1^)	13.5 ± 0.8	18.2 ± 1.2	24.9 ± 1.4
VFAs total (g·L^− 1^)	12.9 ± 0.5	17.6 ± 0.3	28.3 ± 0.9
Lactic acid (g·L^− 1^)	0.5 ± 0.	0.1 ± 0.1	0.3 ± 0.3
Bioconversion (%)	70.1 ± 2.5	64.1 ± 1.8	59.0 ± 1.7
COD_acidified_ (%)	82.3 ± 3.7	76.1 ± 4.0	80.7 ± 2.9

As it can be observed in Table 4, the highest bioconversion efficiency (70.1 ± 2.5%) was reached at the lowest OLR tested (3 g COD·L^-1^·d ^-1^). The bioconversion efficiency decreased as the OLR increased. In UASB-BGS-4.5 and UASB-BGS-6, the bioconversion obtained was 64.1 ± 1.8% and 59.0 ± 1.7%, respectively. This decrease in bioconversion efficiency could be caused by an increase in the concentration of inhibitory compounds present in the substrate (Wainaina et al. 2019b), and by the presence of high concentrations of VFAs (Ramos-Suarez et al. 2021). For instance, Veeken at al. stated that concentration of 30 gCOD·L^-1^ of VFAs could be inhibitory at similar pH ranges used in this study (Veeken et al. 2000). In this case, the concentration of VFAs was 28.8 ± 0.8 gCOD·L^-1^ in UASB-BGS-4.5 and 35.4 ± 1.0 gCOD·L^-1^ in UASB-BGS-6, which can explain the decrease in bioconversion efficiency. On the other hand, similar SCOD/TCOD ratios were determined at increasing OLR (93.4 ± 1.7% in UASB-BGS-3, 90.1 ± 2.9% in UASB-BGS-4.5 and 89.3 ± 2.9% in UASB-BGS-6), demonstrating that the OLR did not have any effect on the hydrolysis step. With regard to the COD_acidified_, no differences were attained neither at increasing OLR. The COD acidified ranged 76–82% (Table 4), these results being higher when compared with the AF of molasses in alternative reactor configurations (65.2% in CSTR and 70.0% in AnSBR) (Lago et al. 2023). Once again, UASB reactors exhibited optimal performance in terms of efficiencies related to carboxylates production.

**Fig. 3:**
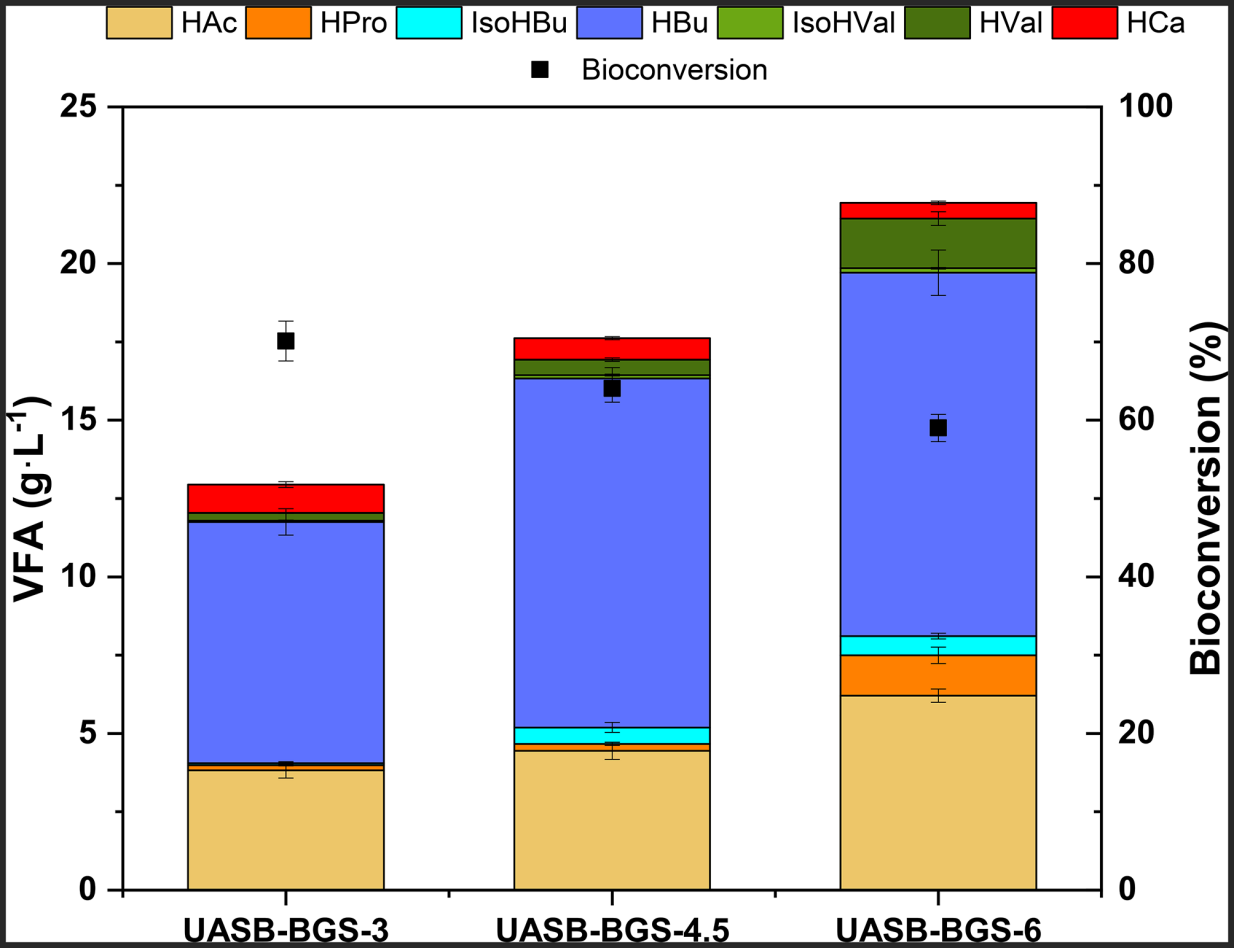
Effect of increasing the OLR in VFAs production, distribution and bioconversions in the UASB using BGS

HBu was the predominant carboxylate regardless of the OLR applied, as it can be seen in Fig. 3. The proportion of HBu was quite similar in UASB-BGS-3 (59.5 ± 2.4%) and in UASB-BGS-4.5 (63.0 ± 1.4%). However, in UASB-BGS-6, HBu concentration decreased to a 52.8 ± 2.2%. The OLR increase did not affect neither to HAc concentration, being the second most abundant carboxylate found in the three OLR tested (29.5 ± 1.7% in UASB-BGS-3, 25.4 ± 1.6% in UASB-BGS-4.5 and 27.6 ± 1.6% in UASB-BGS-6). In this way, it was demonstrated that UASB reactors are a robust reactor configuration able to deal with increasing OLRs without markedly affecting the VFAs distribution.

As a consequence of the OLR increase, TS values in the effluents raised as well from 23.6 ± 1.6 g TS/L in UASB-BGS-3 to 33.8 ± 1.4 g TS/L in UASB-BGS-4.5 and 45.0 ± 1.4 g TS/L in UASB-BGS-6). Even so, TS concentrations remained lower than those achieved with alternative reactor configurations (for instance, CSTRs (Lago et al. 2023). Nevertheless, the OLR increase did not have a negative effect on the g VFA-g TS ratio, being similar for the three OLRs tested (0.55 g VFA/g VS in UASB-BGS-3 and 0.52 g VFA/g VS in UASB-BGS-4.5 and 0.61 g VFA/g VS in UASB-BGS-6). Thereby, the increase in OLR would not affect the VFAs recovery, as the VFAs/VS ratio was similar.

**Fig. 4:**
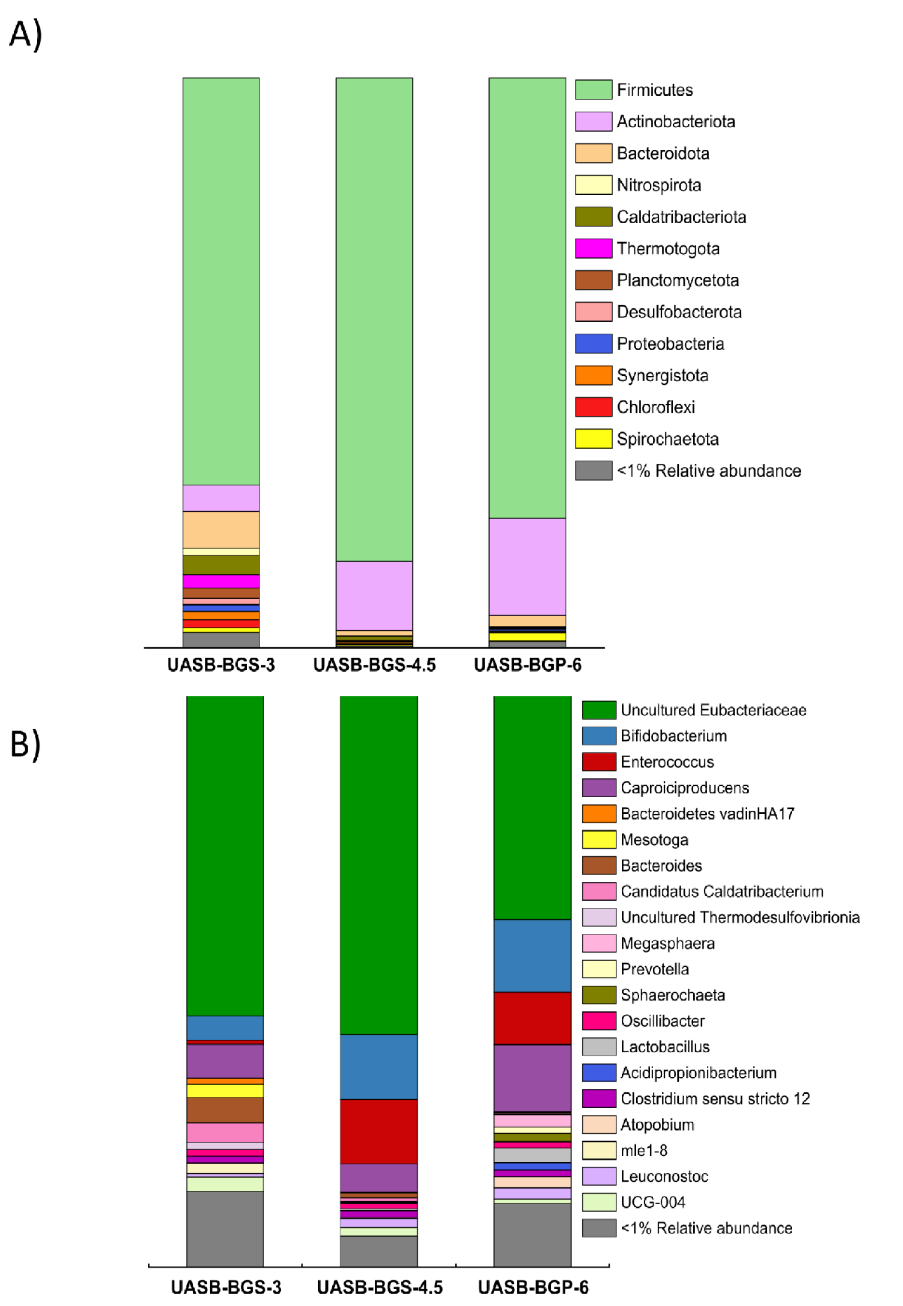
Relative abundance of Bacterial and Archaeal OTUs at phylum (**A**) and genera (**B**) level in BGS inoculum, UASB-BGS-3, UASB-BGS-4.5 and UASB-BGS-6

The abundance of the different microorganisms was also studied to elucidate the effect that an increase in OLR would have on microbial communities. The relative abundance of bacterial and archaeal OTUs at phylum level and at genus level in UASB-BGS-3, UASB-BGS-4.5 and UASB-BGS-6 are presented in Fig. 4.

As a consequence of the imposed operational parameters, the microbial community in UASB-BGS-3 was specialised in VFAs production, especially in HBu. In line with this result, Firmicutes phylum was prevailing. An increase in OLR led to a greater specialization of the microbial community in UASB-BGS-4.5 reactor (Shannon index = 0.56 vs. 1.30 in UASB-BGS-3). The main phyla detected were Firmicutes (84.8%) and Actinobacteria (12.1%), accounting for both phyla 96.9% of the OTUs. Within this phylum, uncultured *Eubacteriaceae* bacteria were once again the most abundant microorganisms, accounting for 59.3% of the total OTUs. As mentioned above, this microbial family has been reported to be strongly related to the HBu production. The increase in Firmicutes was indeed linked to the increase in abundance of *Enterococcus* genus abundance (11.3%). *Enterococcus*, HBu and HAc producers (Radadiya et al. 2023), contributed to the carboxylate’s specialization.

The abundance of Actinobacteria increased concomitantly with OLR. *Bifidobacterium* genus bacteria were the predominant microorganisms (11.3% of total OTUs in UASB-BGS-4.5), promoting the production of lactic acid and HAc in the media. Lactic acid production in UASB-BGS-4.5 was almost negligible (Table 4). Although lactic acid was being produced as a consequence of the presence of this microorganism’s genus, this intermediate metabolite was consumed to produce VFAs, contributing to reach a high acidification degree (76.1 ± 4.0%). Bacteroidota phylum almost disappeared from the media (< 1%), decreasing the production of HPro in UASB-BGS-4.5 reactor.

Increasing the OLR to 6 g COD·L^-1^·d ^-1^ entailed a slight reduction in Firmicutes abundance (77.2%), as well as a decrease in uncultured *Eubacteriaceae* bacteria (39.1%), leading to a slight decrease in HBu concentration compared to UASB-BGS-4.5 (from 63.0 ± 1.4% to 52.8 ± 2.2%). Moreover, *Enterococcus* (HBu producer) abundance decreased to 9.2%. It should be highlighted that at this highest OLR, HVal producers genus such as *Oscillibacter* (1.0%) and *Megasphera* (2.2%) (Yuille et al. 2018) contributed to the increase in HVal production in UASB-BGS-6.

The OLR increase to 6 g COD·L^-1^·d^-1^ increased the relative abundance of Actinobacteria population, reaching a 17% of the total OTUs. *Bifidobacterium* was the main genus of this phylum (12.7% of total OTUs), which has been reported to produce HAc and lactic acid (Lagoa-Costa et al. 2022). Although Bacteroidota was almost negligible in abundance at this OLR, HPro was determined in the cultivation broth. This feature was due to the presence of bacteria from Actinobacteria phylum such as *Acidipropionibacterium* (1.3%), that have also shown HPro production ability (Zheng et al. 2022).

Taking all this into account, it could be concluded that the OLR increase slightly affected the bioconversion efficiencies but not the VFAs distribution profile. The decrease in bioconversion efficiencies was due to the increment of the concentrations of possible inhibitors in the substrate and/or due to the inhibition provoked by high concentration of VFAs reached in high OLR reactors. Despite this, UASB reactors could handle higher OLR to produce VFAs efficiently, allowing the obtaining of effluents with similar solids-to-VFAs ratios.

## Conclusions

This investigation demonstrated that the use of UASB reactors could be an optimal configuration for VFAs production. The utilization of UASB reactors improved effluent characteristics by achieving lower solid content, and enhanced AF performance even under challenging operational conditions, such as low HRT and high OLR, reaching bioconversion of up to 70.1%. Nevertheless, selecting an optimal inoculum source was identified to be crucial to achieve the desired VFAs distribution production. Depending on microbial community composition, the VFA profile could be directed to the production of HBu (concomitantly with higher abundance of Firmicutes phylum) or to HAc and HPro (with an increase in Bacteroidota phylum). The increase in OLR had a slight effect on acidogenic fermentation efficiencies while the VFAs profile distribution still prevailed in HBu (obtaining values from 52.8 to 63.0% w/w of the total VFAs). The microbial community got more specialized at increasing OLR, incrementing the abundance of microorganism from Firmicutes phylum (from 71.4% of the OTUs at a OLR of 3 g_COD_·L^-1^·d^-1^, to a 84.8% and a 77.2% at the OLRs of 4.5 and 6 g_COD_·L^-1^·d^-1^, respectively). Despite those minor changes, the bioconversion efficiencies ranged from 70.1 to 59.0%, which are remarkable values for bioconversion of organic matter to VFAs, proving that UASB reactor is a robust and efficient configuration for acidogenic fermentation.

## Electronic supplementary material

Below is the link to the electronic supplementary material.

Supplementary Material 1

Supplementary Material 2

## Data Availability

The datasets used and/or analysed during the current study are available from the corresponding author on reasonable request and all data generated or analysed during this study are included in this published article.

## Abbreviations

AF: Acidogenic fermentation
AnSBR: Anaerobic sequencing batch reactor
BCP: Biochemical carboxylate potential
BGS: Brewery granular sludge
COD: Chemical oxygen demand
CSTR: Continuous stirred tank reactors
FBR: Fluidised bed reactors
HAc: Acetic acid
HBu: Butyric acid
HCa: Caproic acid
HPro: Propionic acid
HRT: Hydraulic retention time
HVal: Valeric acid
isoHBu: Isobutyric acid
isoHVal: Isovaleric acid
LD: Limit of Detection
N-NH_4_^+^: Ammonium nitrogen
OLR: Organic loading rate
OTUs: Observed operational taxonomic units
PBR: Packed bed reactors
PGS: Paper plant granular sludge
SBR: Sequencing batch reactors
SCOD: Soluble chemical oxygen demand
TCOD: Total chemical oxygen demand
TS: Total Solids
UASB: Up-flow anaerobic sludge blanket
VFAs: Volatile fatty acids
VS: Volatile solids
