# Supplementary material for: Up-flow anaerobic sludge blanket bioreactor for the production of carboxylates: effect of inocula on process performance and microbial communities
Source: Bioresour Bioprocess. 2025 Jan 24;12(1):6. doi: 10.1186/s40643-025-00839-y (PMC11759735; doi:10.1186/s40643-025-00839-y)

Supporting information

**Up-flow anaerobic sludge blanket bioreactor for the production of carboxylates: effect of inocula on process performance and microbial communities**

Adrián Lago^1,2^, Silvia Greses^1,3^, Inés Moreno^2,4^, Cristina González-Fernández^1,5,6*^

^1^Biotechnology Processes Unit, IMDEA Energy, Avda. Ramón de la Sagra 3, 28935, Móstoles, Madrid, Spain.

^2^Thermochemical Processes Unit, IMDEA Energy, Avda. Ramón de la Sagra 3, 28935, Móstoles, Madrid, Spain.

^3^CALAGUA-Unidad Mixta UV-UPV, Departament d’Enginyeria Química, Universitat de València, Avinguda de la Universitat s/n, 46100 Valencia, Spain

^4^Chemical and Environmental Engineering Group, ESCET, Rey Juan Carlos University, 28933, Móstoles, Madrid, Spain

^5^Department of Chemical Engineering and Environmental Technology, School of Industrial Engineering, University of Valladolid, Dr. Mergelina, s/n, Valladolid, 47011, Spain

^6^Institute of Sustainable Processes, Dr. Mergelina, s/n, Valladolid, 47011, Spain

*Corresponding author: email: [cgonfer@uva.es](mailto:cgonfer@uva.es)

Telephone: +34 983 184 596


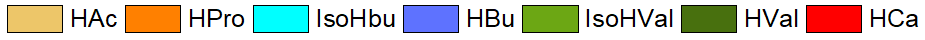


**Figure S1.** Evolution of the concentration of VFAs in BCP test using PGS at 25 ºC (A), at 35 ºC (B) and at 35 ºC (C)

**Figure S2.** Evolution of the concentration of lactic acid and ethanol in BCP test using PGS during the duration of the cycle at 25 ºC (A), at 35 ºC (B) and at 35 ºC (C)


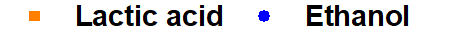

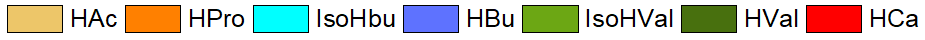


**Figure S3.** Evolution of the concentration of VFAs in BCP test using BGS during the duration of the cycle at 25 ºC (A), at 35 ºC (B) and at 35 ºC (C)

**Figure S4.** Evolution of the concentration of lactic acid and ethanol in BCP test using BGS during the duration of the cycle at 25 ºC (A), at 35 ºC (B) and at 35 ºC (C)


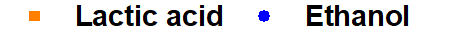

Supplement: Supplementary file 1 — Supplementary Material 1 [file 40643_2025_839_MOESM1_ESM.docx]
