# Supplementary figures and images for: Up-flow anaerobic sludge blanket bioreactor for the production of carboxylates: effect of inocula on process performance and microbial communities
Source: Bioresour Bioprocess. 2025 Jan 24;12(1):6. doi: 10.1186/s40643-025-00839-y (PMC11759735; doi:10.1186/s40643-025-00839-y)

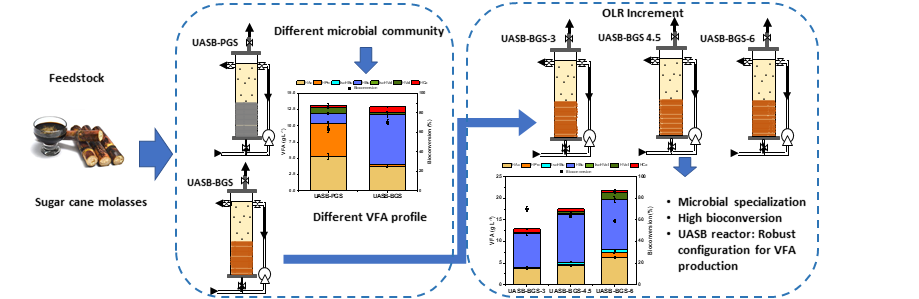

Supplement: Supplementary file 2 — Supplementary Material 2 [file 40643_2025_839_MOESM2_ESM.png]
